# Circulating microRNAs Capture Inflammatory and Muscle Wasting Phenotypes in Patients with Gastrointestinal Cancer

**DOI:** 10.3390/cancers18213509

**Published:** 2026-10-31

**Authors:** Federica Tambaro, Simona Orlando, Giovanni Imbimbo, Carmen Gallicchio, Veronica Rizzo, Elena Belloni, Giuseppe Nigri, Alessio Molfino, Maurizio Muscaritoli

**Affiliations:** 1 Department of Translational and Precision Medicine, Sapienza University of Rome, 00185 Rome, Italy; federica.tambaro@uniroma1.it (F.T.);; 2 Department of Radiological, Oncological and Pathological Sciences, Sapienza University of Rome, 00185 Rome, Italy; veronica.rizzo@uniroma1.it; 3 Department of Medical-Surgical Sciences and Translational Medicine, Sapienza University of Rome, 00185 Rome, Italy; elena.belloni@uniroma1.it (E.B.);; 4 Department of Interdisciplinary Studies on WellBeing, Health and Environmental Sustainability (BeSSA), Sapienza University of Rome, 00185 Rome, Italy

**Keywords:** cancer cachexia, circulating microRNAs, skeletal muscle wasting, systemic inflammation, body composition, gastrointestinal cancer

## Abstract

**Background:** Cancer cachexia (CC) is a multifactorial syndrome marked by systemic inflammation and skeletal muscle (SM) wasting. Circulating microRNAs (miRNAs) may regulate tumor–host interactions. This study investigated whether circulating miRNAs are associated with inflammatory cytokines, CC, and CT-derived SM mass in treatment-naïve patients with gastrointestinal cancer. **Methods**: Fifty-four newly diagnosed patients with gastrointestinal cancer and 15 controls were studied. CC was defined according to Fearon criteria. SM mass was assessed using the CT-derived L3 SM index. Plasma levels of miR-15b, -21, -29a, -29b, -133a, -206, and -486 were quantified by RT-qPCR. Serum concentrations of IL-6, IL-8, IL-10, and TNF-α were measured with a multiplex assay. **Results**: Compared to controls, patients had higher miR-15b (*p* = 0.003), miR-21 (*p* < 0.001), and miR-29b (*p* < 0.001), whereas miR-133a (*p* = 0.007), miR-206 (*p* = 0.022), and miR-486 (*p* = 0.001) were lower. Serum IL-6, IL-8, IL-10, and TNF-α were all elevated in patients vs controls (all *p* < 0.001). miR-29b was higher in cachectic vs non-cachectic patients (*p* = 0.003) and remained independently associated with CC (*p* = 0.007; OR = 10.926; 95% CI: 1.933–61.756). miR-133a was reduced in patients with low SM mass vs. controls (*p* = 0.007). miR-29b was higher in patients with low SM mass and CC vs. those with high SM mass without CC (*p* = 0.026). miR-133a (*p* = 0.004) and miR-206 (*p* = 0.002) were reduced in patients with low SM mass and CC vs. controls. **Conclusions**: Circulating miRNAs showed distinct associations with systemic inflammation, CC, and SM status in treatment-naïve gastrointestinal cancer. These findings support the concept that miRNA alterations reflect distinct biological dimensions of tumor–host interactions and warrant validation in larger longitudinal studies.

## Simple Summary

Cancer cachexia is a complex condition characterized by involuntary weight loss and skeletal muscle wasting that frequently affects patients with cancer. Inflammation and changes in circulating microRNAs, small molecules involved in the regulation of gene expression, may contribute to the biological processes underlying this condition. In this study, we examined circulating microRNAs, inflammatory factors, and skeletal muscle mass in patients with newly diagnosed gastrointestinal cancer before treatment. We found that specific microRNAs were associated with different aspects of cancer-related wasting. In particular, higher circulating miR-29b levels were associated with cancer cachexia, whereas lower miR-133a and miR-206 levels were mainly observed in patients with reduced skeletal muscle mass. These findings suggest that circulating microRNAs may reflect distinct aspects of the interaction between cancer, systemic inflammation, and muscle wasting and provide a basis for further investigation in larger longitudinal studies.

## 1. Introduction

Cancer cachexia (CC) is a multifactorial wasting syndrome characterized by involuntary skeletal muscle (SM) loss, with or without adipose tissue depletion, that cannot be fully reversed by conventional nutritional support and leads to progressive functional impairment [1]. Rather than a mere consequence of reduced food intake, CC reflects a complex tumor–host interaction driven by systemic inflammation, metabolic derangements, neurohormonal alterations, and tissue-specific catabolic remodeling [2]. CC affects a substantial proportion of patients with advanced cancer and is associated with impaired functional status, reduced treatment tolerance, poorer quality of life, and increased mortality [3]. Given the detrimental impact of CC on clinical outcomes, increasing attention has been directed toward the biological mechanisms that sustain cancer-associated wasting and drive the progressive deterioration of SM mass.

SM wasting is the central phenotypic feature of CC and reflects a sustained imbalance between anabolic and catabolic pathways, involving altered protein turnover, mitochondrial dysfunction, impaired regenerative capacity, and metabolic reprogramming [4]. These alterations are closely linked to systemic inflammation, which contributes to the activation of catabolic signaling and the suppression of anabolic and reparative responses in SM [5,6]. Nevertheless, inflammation alone does not fully explain the heterogeneity of CC phenotypes. Patients with similar tumor types or comparable degrees of weight loss may show different patterns of muscle depletion, nutritional impairment, and systemic metabolic derangement. This variability suggests that additional regulatory layers shape the host response to cancer. Among them, microRNAs (miRNAs) have attracted increasing attention because they can modulate multiple gene networks simultaneously and influence biological processes directly relevant to wasting, including myogenesis, proteostasis, mitochondrial metabolism, inflammatory signaling, adipose tissue remodeling, and intercellular communication [7]. In this sense, miRNAs may be active components of the regulatory circuits through which cancer and inflammation affect peripheral tissues. This concept is supported by experimental and clinical evidence showing that selected miRNAs can participate in muscle atrophy and cachexia-related pathways [8]. Previous work from our group has contributed to this framework by showing that small non-coding RNA alterations in gastrointestinal cancer are detectable both at the systemic level and within tissues affected by body composition changes, namely SM and adipose tissue [9,10,11]. In particular, these studies suggest that dysregulation of non-coding RNAs may be part of the molecular architecture linking cancer, inflammation, and host tissue wasting. However, the relationship as well as the relevant mechanistic implication between circulating miRNA patterns, systemic inflammation, cachexia status, and SM impairment in cancer remain incompletely defined. Clarifying these relationships may help better understand whether selected miRNAs align with the inflammatory and SM-depleting phenotype of CC, rather than representing isolated molecular alterations.

The present study aimed to investigate a panel of circulating miRNAs implicated in SM homeostasis, inflammatory regulation, and cancer-associated tissue remodeling in treatment-naïve patients with gastrointestinal cancer. We further explored their associations with circulating cytokines, cachexia status, and SM impairment. By integrating these domains, this work sought to explore whether distinct miRNAs map onto different dimensions of cancer-associated wasting.

## 2. Materials and Methods

### 2.1. Study Design and Patient Enrollment

This was a single-center observational study of consecutive patients with gastrointestinal cancers (gastric, pancreatic, and colorectal) referred to the Department of Medical-Surgical Sciences and Translational Medicine at Sapienza University of Rome, Italy. Laboratory and molecular analyses were performed at the Department of Translational and Precision Medicine at the same university.

Eligible patients were enrolled at the time of diagnosis, before any surgical or oncological treatment, among those scheduled for surgical tumor resection. Inclusion criteria included age ≥18 years, histologically confirmed gastrointestinal cancer diagnosed within the previous four weeks, and no prior exposure to anticancer, corticosteroid, or anti-inflammatory treatments. Patients were excluded if they were younger than 18 years or had concomitant conditions known to interfere with nutritional status or systemic inflammation, including active infections, hepatic or renal failure, heart failure, autoimmune diseases, severe malabsorption syndromes, intestinal obstruction, dysphagia, or other chronic inflammatory conditions.

The control group included subjects scheduled for elective surgery for non-malignant conditions, with no history of malignancy or inflammatory diseases, including cholecystectomy for gallstones (*n* = 4), abdominal wall surgery for hernia (*n* = 6) and mesenteric cyst excision (*n* = 5).

This study was conducted in accordance with the Declaration of Helsinki and approved by the Local Ethics Committee of Sapienza University, Azienda Sant’Andrea Hospital, Rome, Italy (protocol number 167_2017).

All participants provided written informed consent before study participation.

### 2.2. Clinical and Nutritional Assessment

At enrollment, all participants underwent a comprehensive clinical and nutritional evaluation. For each subject, demographic characteristics, medical history, comorbidities, tumor stage, and histological diagnosis were recorded.

Anthropometric assessment included measurements of body weight (kg) and height (m), which were used to calculate body mass index (BMI, kg/m^2^).

CC was diagnosed according to Fearon’s criteria [1], and patients were classified as cachectic if weight loss was >5% over the previous 6 months. Weight loss history was obtained from patient-reported information collected during the baseline clinical assessment.

### 2.3. Assessment of Body Composition

Skeletal muscle mass was evaluated using abdominal computed tomography (CT) images obtained for routine clinical purposes. For each patient, a single axial image at the third lumbar vertebra (L3) level was selected, and total skeletal muscle area (SMA) was quantified by including the *psoas*, paraspinal, and abdominal wall muscle groups [9]. Images were analyzed using OsiriX Lite DICOM Viewer (version 11.0.3, Pixmeo SARL, Bernex, Switzerland), as previously described [10]. L3 skeletal muscle index (L3-SMI, cm^2^/m^2^) was calculated by dividing SMA by height squared.

Low muscle mass (LMM) was defined using internal sex-specific cut-offs based on the median L3-SMI value, corresponding to 47.89 cm^2^/m^2^ for males and 39.14 cm^2^/m^2^ for females. CT-derived skeletal muscle assessment was available for 44 of the 54 participants. The remaining 10 patients were excluded from the body composition analyses because CT images were unavailable.

### 2.4. Blood Collection and Sample Processing

At study enrollment, blood samples were collected from all participants before the scheduled surgical procedure. For serum preparation, fasting peripheral blood was collected into additive-free Vacutainer^®^ Serum tubes (Becton, Dickinson and Company, Franklin Lakes, NJ, USA) and allowed to clot at room temperature for at least 30 min. Samples were then centrifuged at 1500× *g* for 10 min at 4 °C, aliquoted, and the resulting serum was stored at −80 °C until use. For plasma isolation, fasting peripheral blood samples were collected in EDTA-containing tubes and processed within 2 h. After centrifugation at 3000 rpm for 15 min at 4 °C, plasma was separated, aliquoted, and stored at −80 °C until further analysis.

### 2.5. RNA Isolation and Quantitative Real-Time PCR

For circulating miRNA analysis, total RNA was extracted from plasma samples using the RNeasy Serum/Plasma Kit (Qiagen, Germantown, MD, USA) following the manufacturer’s instructions. A 3 µL amount of cel-miR-39-3p synthetic miRNA spike-in control (Applied Biosystems, Thermo Fisher Scientific, Waltham, MA, USA) was added after lysis reagent to evaluate RNA extraction efficiency. RNA concentration and purity were measured with the Multiskan Sky spectrophotometer (Thermo Fisher Scientific, Waltham, MA, USA). cDNA was synthesized from 10 ng of total RNA using the TaqMan Advanced miRNA cDNA Synthesis kit (Applied Biosystems, Thermo Fisher Scientific, Waltham, MA, USA), according to the manufacturer’s protocol. Quantitative real-time PCR was carried out with TaqMan Fast Advanced Master Mix (Applied Biosystems, Thermo Fisher Scientific, Waltham, MA, USA) on a QuantStudio^®^ Real-Time PCR system (Applied Biosystems, Thermo Fisher Scientific, Waltham, MA, USA). Target miRNAs were quantified using specific TaqMan miRNA Assay probes (A25576, Applied Biosystems, Thermo Fisher Scientific, Waltham, MA, USA) for hsa-miR-15b-3p, hsa-miR-21-5p, hsa-miR-29a-3p, hsa-miR-29b-3p, hsa-miR-133a-3p, hsa-miR-206, and hsa-miR-486-5p. Expression data were normalized to hsa-miR-16-5p, which was chosen as the endogenous control after confirming its stability. Relative miRNA levels were determined from two independent qPCR experiments, each in duplicate, with an internal calibrator to control for inter-run variability. Raw amplification data were analyzed using SDS2.4 software (version 2.4, Applied Biosystems, Bedford, MA, USA), and relative expression was calculated using the 2^−ΔΔCt^ method.

### 2.6. Serum Cytokine Multiplex Assay

Serum levels of IL-6, IL-8, IL-10, and TNF-α were quantified with the custom human premixed multiplex magnetic Luminex^®^ assay (cat. #LXSAHM-04, Bio-Techne, San Francisco, CA, USA). All steps followed the manufacturer’s guidelines. Briefly, serum samples were diluted 1:1 (*v/v*) in assay buffer, then added to a 96-well plate with the microparticle cocktail and incubated overnight at 4 °C. After washing, biotinylated detection antibodies were added for 1 h at room temperature, followed by Streptavidin-PE for 30 min at room temperature. Measurements were performed in duplicate using Luminex^®^ xMAP^®^ technology (Luminex Corp., Austin, TX, USA). An intra-assay CV of 20% was used as a cutoff, with all duplicates within this range. Assay recovery ranged from 80% to 120%. For each analyte, an eight-point standard curve was created using a five-parameter logistic (5-PL) model. Data was collected with xPONENT software (version 4.3, Luminex Corp., Austin, TX, USA) and analyzed with Quantist Luminex^®^ Data Analysis Software (version 2.0, Bio-Techne, San Francisco, CA, USA). Final cytokine levels are reported in pg/mL.

### 2.7. Statistical Analyses

Data are shown as mean ± standard deviation (SD) or median with interquartile range (IQR) for normally or non-normally distributed variables, respectively. Graphical data are presented as mean ± standard error of the mean (SEM), unless otherwise indicated. Categorical variables are given as counts and percentages. Before analysis, data distribution was checked to determine whether to use parametric or nonparametric tests. For continuous variables, group comparisons were made using the unpaired Student’s *t*-test or the Mann–Whitney U test, based on distribution. For three or more groups, one-way ANOVA was used for normally distributed data, and the Kruskal–Wallis test for non-normal data. Categorical variables were compared with the chi-square or Fisher’s exact test, as appropriate. When overall differences were found in multi-group analysis, Tukey’s or Dunn’s post hoc tests were used after one-way ANOVA or Kruskal–Wallis, respectively.

Spearman’s correlation coefficient (r) was employed to examine relationships between circulating miRNA levels, cytokines, and clinical and nutritional parameters. A multivariable logistic regression was conducted to identify variables independently linked to cancer cachexia. Variables for this model were chosen based on their biological significance and/or notable findings in univariate analyses. The final model incorporated age, gender, tumor type, and circulating miR-29b levels. Tumor type was categorized as non-colorectal versus colorectal cancer. The results from the logistic regression are presented as the B coefficient, odds ratio (OR), 95% confidence interval (CI), and *p*-value. A two-tailed *p*-value < 0.05 was deemed statistically significant. All statistical analyses were carried out using IBM^®^ SPSS Statistics version 30.0 (SPSS Inc., Chicago, IL, USA) and GraphPad Prism 10.2.3 (San Diego, California, USA).

## 3. Results

### 3.1. Patients’ Characteristics

This study included 54 patients with gastrointestinal cancer and 15 control subjects. Clinical and demographic characteristics of the study population are summarized in Table 1.

Among patients with gastrointestinal cancer, 39% were female, with a median age of 73 years (64; 78). Among controls, 47% were female, with a median age of 54 years (51; 66). Based on the Fearon criteria [1], 24 patients (44%) were identified as cachectic. For cancer patients, CT-derived body composition data were available, showing a mean SMA of 113.7 ± 40.7 cm^2^ and sex-specific mean L3-SMI values of 50.98 ± 8.1 cm^2^/m^2^ in males and 39.44 ± 6.6 cm^2^/m^2^ in females. The most frequent tumor site was colorectal cancer (87%), followed by gastric (7%) and pancreatic cancer (6%). Overall, 61% of cancer patients had stage I-II disease, while 39% had stage III-IV.

### 3.2. Circulating miRNA Levels and Inflammatory Cytokine Profile in Gastrointestinal Cancer and Controls

**Figure 1:**
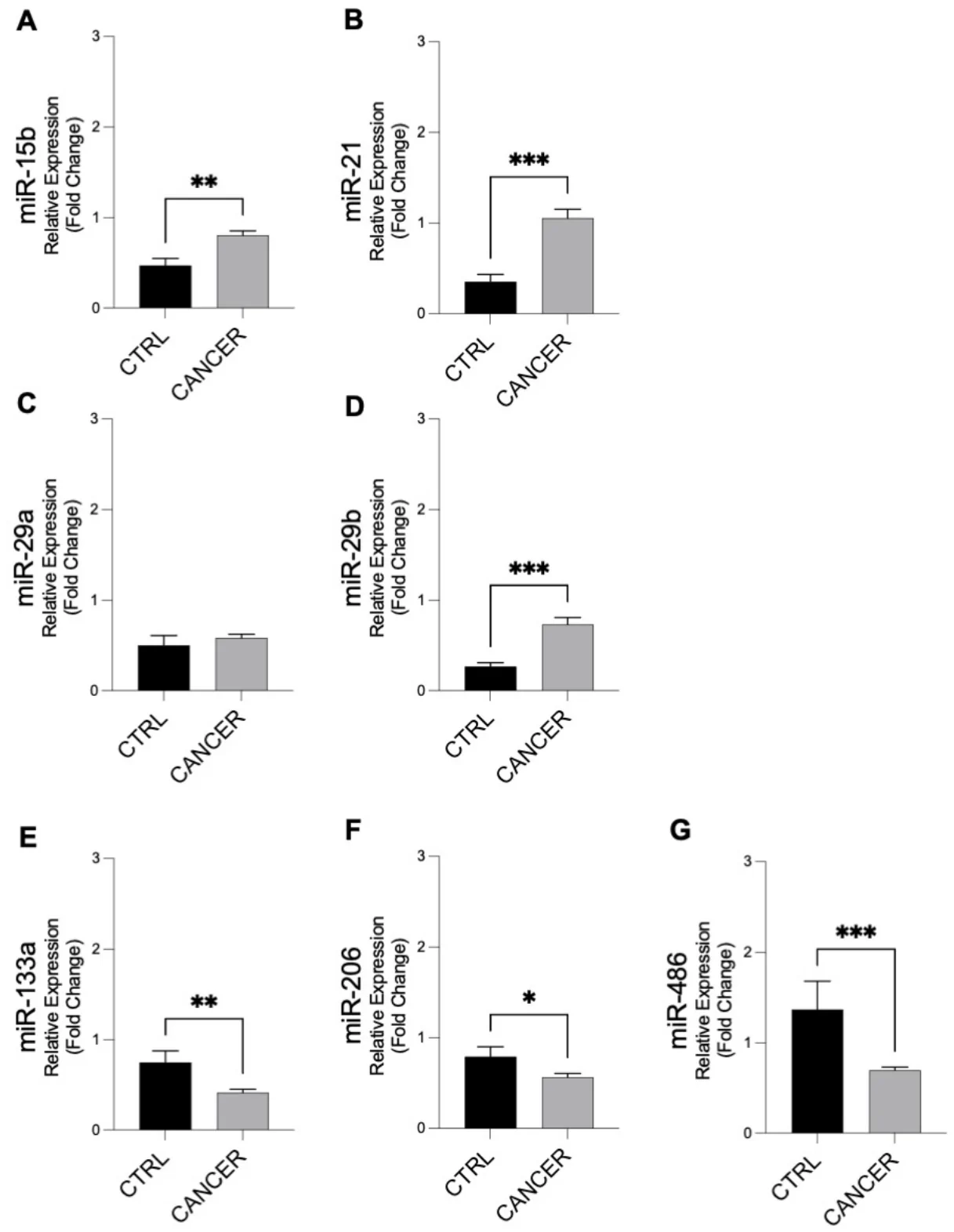
Differences in circulating miRNAs between patients with gastrointestinal cancer and controls. Circulating miRNA levels were quantified by quantitative real-time PCR in patients with gastrointestinal cancer (*n* = 54) and controls (*n* = 15). Data show significantly increased levels of miR-15b (*p* = 0.003) (**A**), miR-21 (*p* < 0.001) (**B**), and miR-29b (*p* < 0.001) (**D**), and significantly lower levels of miR-133a (*p* = 0.007) (**E**), miR-206 (*p* = 0.022) (**F**), and miR-486 (*p* = 0.001) (**G**) in patients with gastrointestinal cancer compared to controls. No significant difference was observed in miR-29a levels (**C**). Data are expressed as fold change and presented as mean ± SEM. Student’s *t*-test and the Mann–Whitney test were used for normally distributed or non-normally distributed data, respectively. * *p* ≤ 0.05, ** *p* ≤ 0.01, *** *p* ≤ 0.001.

To characterize the circulating miRNA profile linked to gastrointestinal cancer, plasma levels of selected miRNAs were measured using RT-qPCR and compared between patients and controls (Figure 1).

Patients with gastrointestinal cancer showed significantly increased circulating levels of miR-15b (*p* = 0.003), miR-21 (*p* < 0.001), and miR-29b (*p* < 0.001) compared to controls (Figure 1A,B,D). In contrast, miR-133a (*p* = 0.007), miR-206 (*p* = 0.022), and miR-486 (*p* = 0.001) were noticeably lower in the cancer group than in controls (Figure 1E–G). There was no significant difference in miR-29a levels between the groups (Figure 1C).

**Figure 2:**
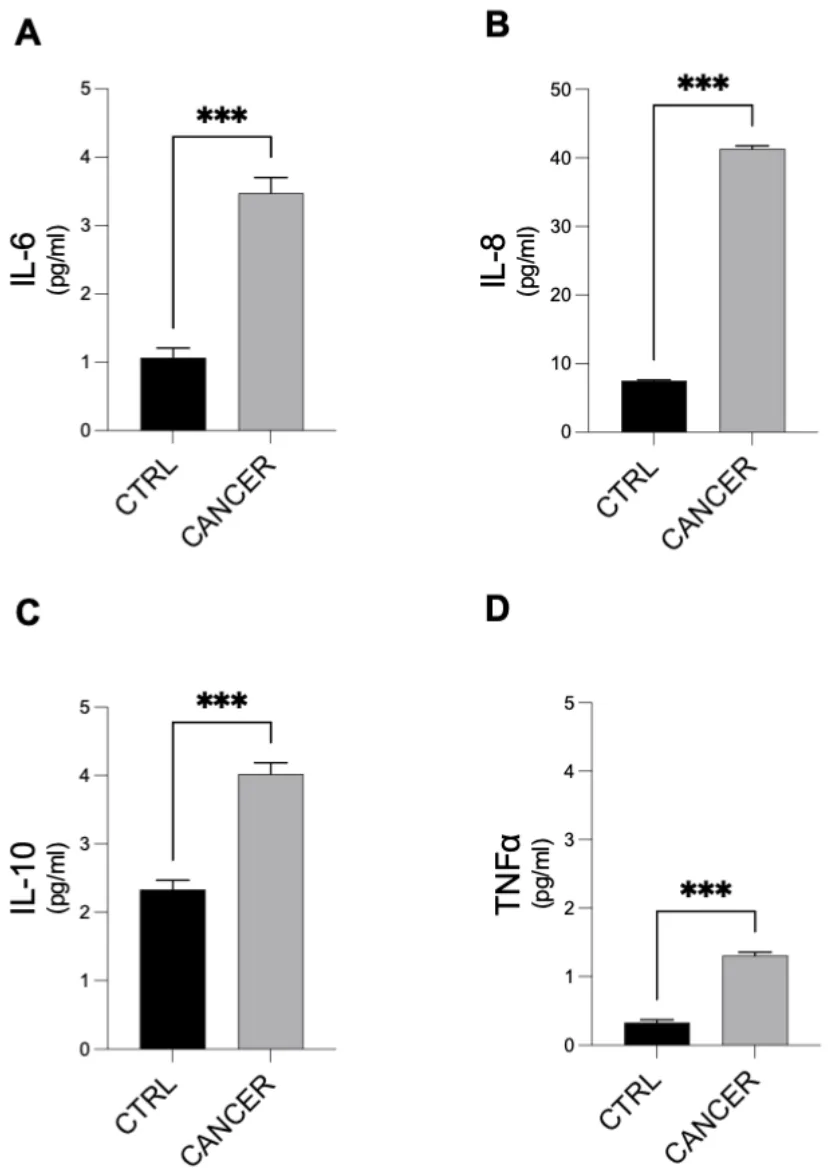
Circulating cytokine profile in patients with gastrointestinal cancer and controls. Circulating cytokine concentrations were measured using a custom human premixed multiplex magnetic Luminex^®^ assay in patients with gastrointestinal cancer (*n* = 54) and controls (*n* = 15). Patients with gastrointestinal cancer exhibited markedly higher circulating concentrations of IL-6 (*p* < 0.001) (**A**), IL-8 (*p* < 0.001) (**B**), IL-10 (*p* < 0.001) (**C**), and TNF-α (*p* < 0.001) (**D**) than controls. Data are presented as mean ± SEM. Student’s *t*-test and the Mann–Whitney test were used for normally distributed or non-normally distributed data, respectively. *** *p* ≤ 0.001.

We then evaluated whether gastrointestinal cancer was associated with systemic inflammatory changes using xMAP technology. Circulating concentrations of IL-6, IL-8, IL-10, and TNF-α were markedly higher in patients with gastrointestinal cancer than in controls, with all differences reached statistical significance (all *p* < 0.001) (Figure 2).

To explore the relationship between circulating miRNAs and systemic inflammatory mediators, Spearman’s correlation analyses were performed (Supplementary Table S1). Among miRNAs increased in patients with gastrointestinal cancer, miR-15b was positively correlated with IL-10 (r = 0.383, *p* = 0.001) and TNF-α (r = 0.347, *p* = 0.003), whereas no significant correlations were observed with IL-6 or IL-8. miR-21 was positively correlated with IL-6 (r = 0.330, *p* = 0.006), IL-8 (r = 0.226, *p* = 0.017), and TNF-α (r = 0.298, *p* = 0.013), whereas no significant correlation was observed with IL-10. miR-29b was positively correlated with IL-6 (r = 0.413, *p* < 0.001), IL-8 (r = 0.403, *p* < 0.001), IL-10 (r = 0.277, *p* = 0.021), and TNF-α (r = 0.259, *p* = 0.032). miR-29a did not show significant correlations with any of the analyzed cytokines (Supplementary Table S1).

Among miRNAs reduced in patients with gastrointestinal cancer, miR-133a was negatively correlated with IL-6 (r = −0.301, *p* = 0.012), IL-8 (r = −0.276, *p* = 0.022), and IL-10 (r = −0.383, *p* = 0.001), whereas no significant correlation was observed with TNF-α. miR-206 was negatively correlated with IL-6 (r = −0.334, *p* = 0.05) and IL-10 (r = −0.288, *p* = 0.016), whereas no significant correlations were observed with IL-8 or TNF-α. miR-486 was negatively correlated with IL-8 (r = −0.266, *p* = 0.027), whereas no significant correlations were observed with IL-6, IL-10, or TNF-α (Supplementary Table S1).

### 3.3. Circulating miRNA Alterations Across Gastrointestinal Tumor Types

In this exploratory analysis, we assessed whether circulating miRNA levels varied by tumor site. We categorized gastrointestinal cancer patients into colorectal, gastric, and pancreatic groups and compared them with controls (Supplementary Figure S1). Due to the small number of gastric and pancreatic cancer cases, these findings should be considered exploratory and purely descriptive.

Compared to controls, patients with colorectal cancer showed significantly higher miR-15b levels (*p* = 0.006), as did patients with gastric cancer (*p* = 0.009) (Supplementary Figure S1A). miR-21 levels were markedly elevated in patients with colorectal (*p* < 0.001), gastric (*p* = 0.010), and pancreatic cancers (*p* < 0.001), with a significant difference also seen between colorectal and pancreatic cancer patients (*p* = 0.022) (Supplementary Figure S1B). Levels of miR-29b were increased across all three tumor groups compared to control group, with significantly higher levels in colorectal (*p* < 0.001), gastric (*p* = 0.015), and pancreatic cancer patients (*p* < 0.001), and a difference was observed between colorectal and pancreatic cancer groups (*p* = 0.017) (Supplementary Figure S1D). miR-133a was diminished in patients with colorectal (*p* = 0.017) and gastric cancer (*p* = 0.007) compared to controls (Supplementary Figure S1E). miR-206 levels were significantly lower in patients with gastric (*p* = 0.001) and pancreatic cancer (*p* = 0.037), with a notable difference between these two groups (*p* = 0.014) (Supplementary Figure S1F). miR-486 was significantly decreased in colorectal (*p* = 0.002) and pancreatic cancer patients (*p* = 0.009), but not gastric cancer patients, compared to controls (Supplementary Figure S1G). No significant differences were found in miR-29a levels (Supplementary Figure S1C).

### 3.4. Association of Circulating miRNAs with Cancer Cachexia

**Figure 3:**
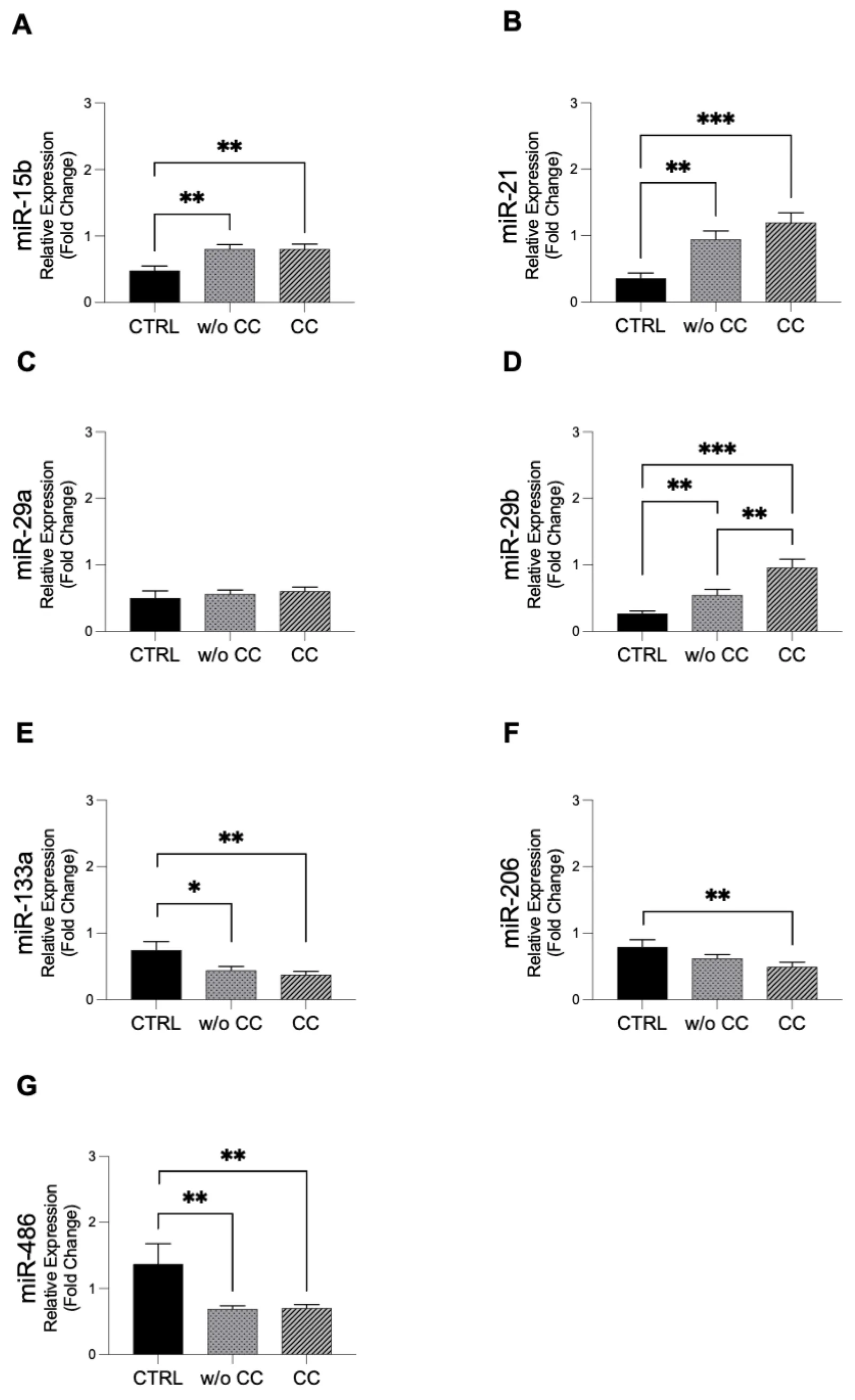
Differences in circulating plasma miRNAs among cancer patients and controls. Circulating miRNA levels were analyzed in gastrointestinal cancer patients with cancer cachexia (*n* = 24) and without cancer cachexia (*n* = 30), and in a control group (*n* = 15). (**A**,**B**) miR-15b and miR-21 levels were significantly increased in both CC (*p* = 0.009 and *p* < 0.001, respectively) and w/o CC (*p* = 0.007 and *p* = 0.002, respectively) patients compared with controls. (**D**) miR-29b levels were significantly increased in both CC (*p* < 0.001) and w/o CC (*p* = 0.006) patients compared with controls, with significantly higher levels in CC than in w/o CC patients (*p* = 0.003). (**E**,**G**) miR-133a and miR-486 levels were significantly reduced in both CC (*p* = 0.005 and *p* = 0.004, respectively) and w/o CC (*p* = 0.033 and *p* = 0.002, respectively) patients compared with controls. (**F**) miR-206 levels were significantly decreased in CC patients compared with controls (*p* = 0.008). (**C**) No significant differences were observed for miR-29a. Data are expressed as fold change and presented as mean ± SEM. One-way ANOVA or the Kruskal–Wallis test was used for normally distributed or non-normally distributed data, respectively. * *p* ≤ 0.05, ** *p* ≤ 0.01, *** *p* ≤ 0.001. Abbreviations: CC, cancer cachexia; w/o CC, without cancer cachexia; ctrl, control group.

To assess whether circulating miRNA alterations were associated with nutritional impairment, patients with gastrointestinal cancer were stratified based on the presence or absence of CC (Figure 3).

Notably, among the investigated miRNAs, only miR-29b showed significantly higher levels in cachectic compared with non-cachectic patients, whereas the other miRNA alterations observed were irrespective of cachexia status. miR-29b levels were significantly higher in cachectic than in non-cachectic patients (*p* = 0.003) and were increased in both cachectic (*p* < 0.001) and non-cachectic patients (*p* = 0.006) compared with controls (Figure 3D). In the same stratification, miR-15b and miR-21 levels were significantly increased in patients with CC (*p* = 0.009 and *p* < 0.001, respectively) and in those without CC (*p* = 0.007 and *p* = 0.002, respectively) compared with controls (Figure 3A,B). miR-133a and miR-486 levels were significantly reduced in cachectic (*p* = 0.005 and *p* = 0.004, respectively) and non-cachectic patients (*p* = 0.033 and *p* = 0.002, respectively) compared with controls (Figure 3E,G). miR-206 levels were reduced in cachectic patients compared with controls (*p* = 0.008), whereas no significant difference was observed between non-cachectic patients and controls (Figure 3F). No significant differences were observed for miR-29a when considering the presence or absence of CC in our cohort (Figure 3C).

Considering the difference observed between cachectic and non-cachectic patients, we performed a multivariable logistic regression to determine if circulating miR-29b was independently linked to the cachectic phenotype after controlling for age, sex, and tumor type (Table 2).

The analysis showed that circulating miR-29b remained significantly associated with cancer cachexia (*p* = 0.007; OR = 10.926; 95% CI: 1.933–61.756), while age, sex, and tumor type did not show significant associations (Table 2).

### 3.5. Circulating miRNAs According to Skeletal Muscle Mass and Cachexia

**Figure 4:**
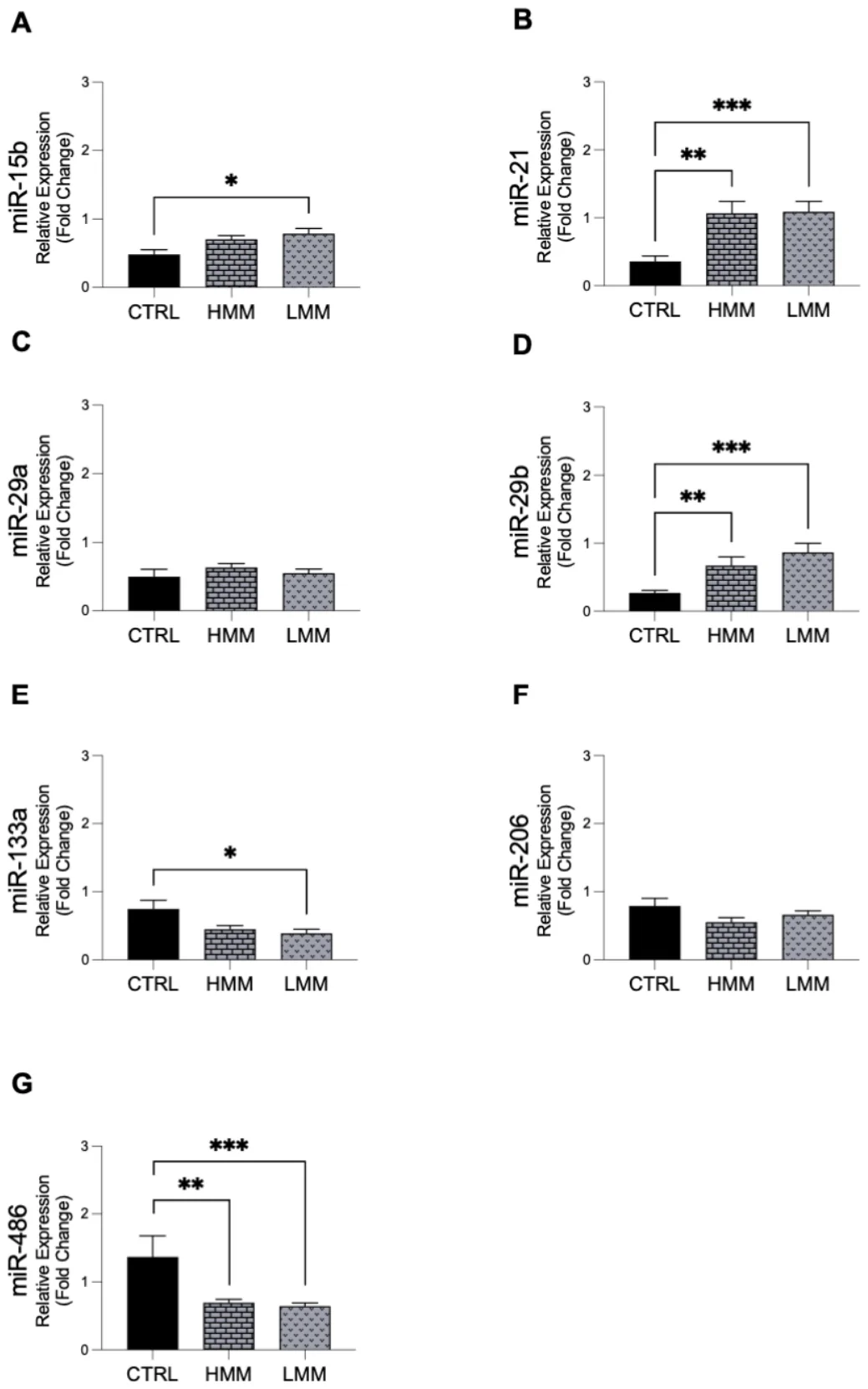
Circulating miRNA profile according to skeletal muscle mass in patients with gastrointestinal cancer and controls. Circulating miRNA levels were analyzed in patients with gastrointestinal cancer, stratified by skeletal muscle mass assessed by CT using sex-specific internal L3-SMI cutoffs (median values: 47.89 cm^2^/m^2^ for males and 39.14 cm^2^/m^2^ for females). Patients were classified as having low muscle mass (*n* = 23) or high muscle mass (*n* = 21) and compared with controls (*n* = 15). The analysis included 44 patients with available CT-derived skeletal muscle mass data. (**A**) miR-15b levels were significantly higher in LMM patients than in controls (*p* = 0.011). (**B**,**D**) miR-21 and miR-29b levels were significantly higher in both LMM (*p* < 0.001 for both) and HMM (*p* < 0.001 and *p* = 0.002, respectively) patients than in controls. (**E**,**G**) miR-133a and miR-486 levels were significantly lower in LMM patients than in controls (*p* = 0.007 and *p* < 0.001, respectively), and miR-486 levels were also significantly lower in HMM patients vs. controls (*p* = 0.003). (**C**,**F**) No significant differences were observed for miR-29a and miR-206. Data are expressed as fold change and presented as mean ± SEM. One-way ANOVA and the Kruskal–Wallis test were used for normally distributed or non-normally distributed data, respectively. * *p* ≤ 0.05, ** *p* ≤ 0.01, *** *p* ≤ 0.001. Abbreviations: LMM, low muscle mass; HMM, high muscle mass; ctrl, control group.

We next evaluated circulating miRNA levels in relation to CT-derived SM mass (Figure 4).

Patients were classified as having low or high muscle mass using sex-specific internal L3-SMI cut-offs and compared with controls. miR-15b levels were significantly higher in patients with low muscle mass than in controls (*p* = 0.011) (Figure 4A). miR-21 and miR-29b levels were significantly higher in both low-muscle-mass (*p* < 0.001 for both) and high-muscle-mass patients (*p* < 0.001 and *p* = 0.002, respectively) than in controls (Figure 4B,D). miR-133a levels were significantly lower only in patients with low muscle mass than in controls (*p* = 0.007) (Figure 4E), whereas miR-486 levels were significantly lower in both low-muscle-mass (*p* < 0.001) and high-muscle-mass subgroups (*p* = 0.003) than in controls (Figure 4G). No significant differences were observed for miR-29a or miR-206 across SM mass subgroups (Figure 4C,F). Overall, miR-15b and miR-133a showed significant modulations only in patients with low SM mass compared with controls, whereas miR-21, miR-29b, and miR-486 were significantly modulated in both low- and high-SM-mass subgroups.

**Figure 5:**
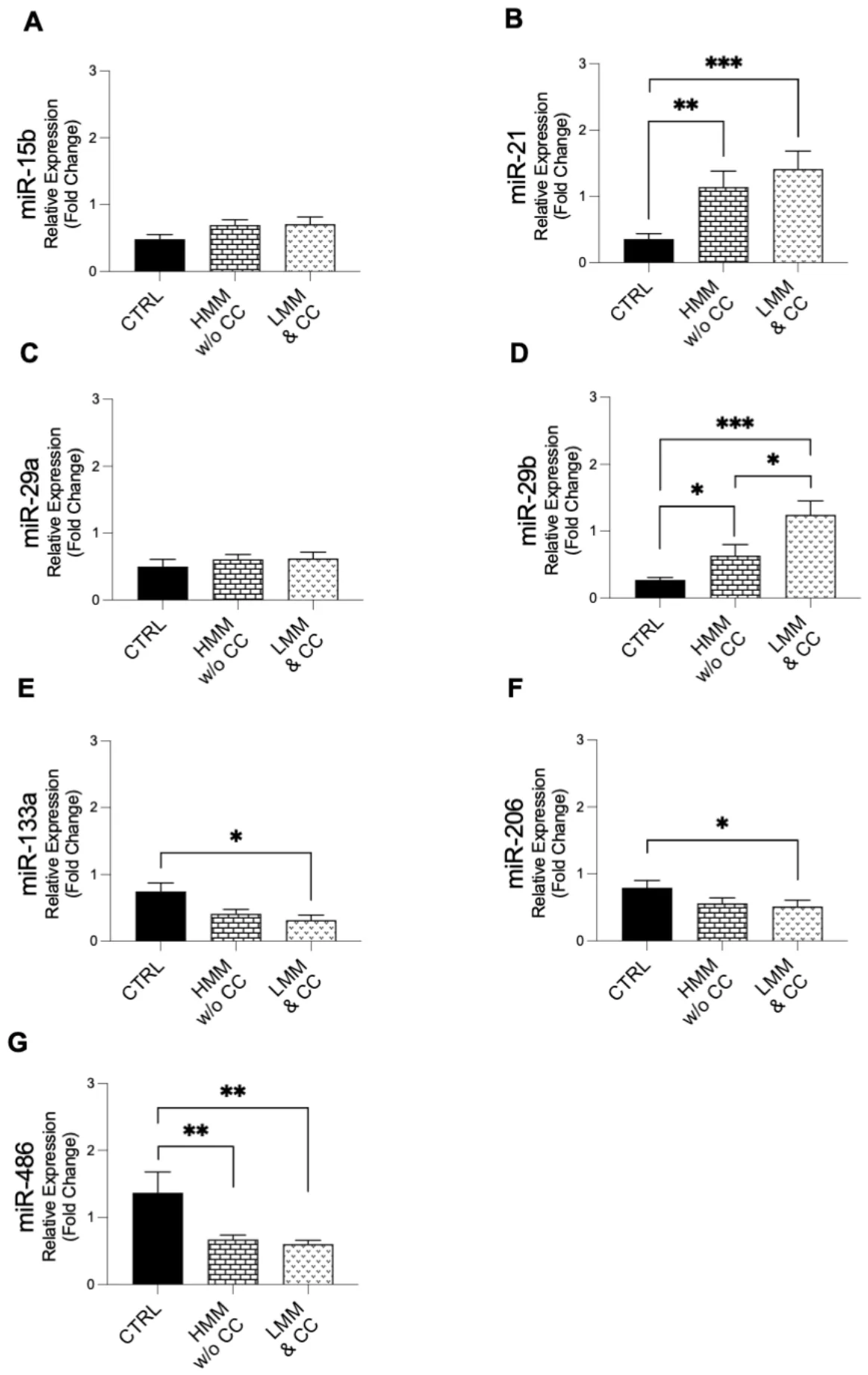
Circulating miRNA profile according to the combined presence of cancer cachexia and skeletal muscle mass in patients with gastrointestinal cancer and controls. Circulating miRNA levels were analyzed in patients with gastrointestinal cancer, stratified by the combined presence or absence of cachexia and muscularity. Patients were divided as having low muscle mass and cancer cachexia (*n* = 11), high muscle mass without cancer cachexia (*n* = 14), and controls (*n* = 15). (**D**) miR-29b levels were significantly higher in LMM+CC compared to HMM without CC patients (*p* = 0.026) and significantly increased in both LMM+CC (*p* < 0.001) and HMM without CC (*p* = 0.019) patients compared with controls. (**B**) miR-21 levels were significantly increased in both LMM+CC (*p* < 0.001) and HMM without CC (*p* = 0.003) patients compared with controls. (**E**,**F**) miR-133a and miR-206 levels were significantly reduced in LMM+CC patients compared with controls (*p* = 0.004 and *p* = 0.002, respectively). (**G**) miR-486 levels were significantly reduced in both LMM+CC (*p* = 0.002) and HMM without CC (*p* = 0.005) patients compared with controls. (**A**,**C**) No significant differences were observed for miR-15b and miR-29a. Data are expressed as fold change and presented as mean ± SEM. One-way ANOVA and the Kruskal–Wallis test were used for normally distributed or non-normally distributed data, respectively. * *p* ≤ 0.05, ** *p* ≤ 0.01, *** *p* ≤ 0.001. Abbreviations: CC, cancer cachexia; LMM, low muscle mass; HMM, high muscle mass; w/o CC, without cancer cachexia; ctrl, control group.

To further characterize the relationship between circulating miRNAs, cachexia, and muscle impairment, patients were stratified according to a combined phenotype including both CC and SM mass. Patients with low muscle mass and CC were compared with patients with high muscle mass without cachexia and controls (Figure 5).

miR-29b levels were significantly higher in low-muscle-mass/cachectic patients than in high-muscle-mass/non-cachectic patients (*p* = 0.026) and were significantly increased in both low-muscle-mass/cachectic patients (*p* < 0.001) and high-muscle-mass/non-cachectic patients (*p* = 0.019) compared with controls (Figure 5D). miR-21 levels were significantly increased in both low-muscle-mass/cachectic patients (*p* < 0.001) and high-muscle-mass/non-cachectic patients (*p* = 0.003) compared with controls (Figure 5B). miR-486 levels were significantly reduced in both low-muscle-mass/cachectic patients (*p* = 0.002) and high-muscle-mass/non-cachectic patients (*p* = 0.005) compared with controls (Figure 5G), whereas miR-133a and miR-206 levels were significantly reduced only in low-muscle-mass/cachectic patients compared with controls (*p* = 0.004 and *p* = 0.002, respectively) (Figure 5E,F). No significant differences were observed for miR-15b or miR-29a among patients with low muscle mass and CC, patients with high muscle mass without CC, and controls (Figure 5A,C). In the combined analysis, miR-29b was the only miRNA showing a significant difference between patients with low SM mass and CC and those with high SM mass without CC, whereas the reductions in miR-133a and miR-206 were significant only in comparisons between the low-SM-mass/CC subgroup and controls.

## 4. Discussion

CC is increasingly recognized as an active, tumor-driven wasting condition sustained by systemic inflammation and tissue-specific catabolic reprogramming across multiple tissues [5]. In this context, circulating miRNAs have emerged as potential mediators of molecular signals that reflect the crosstalk between tumor, immune system, and SM wasting, although the molecular mechanisms remain only partially understood [12].

This study fills a research gap by combining circulating miRNA profiling with measurements of inflammatory cytokines, cachexia status, and CT-based SM assessment in treatment-naïve gastrointestinal cancer patients. While associations between circulating miRNAs, cachexia and inflammation have been previously described [12], to our knowledge these have mostly been investigated separately or in advanced or treated patients. By integrating circulating miRNA and cytokine profiles with cachexia status and CT-derived SM mass in treatment-naïve patients with gastrointestinal cancer, our study provides a novel and more comprehensive characterization of these relationships. Our results suggest a coordinated circulating miRNA signature linked to cancer-related inflammation and wasting already before any anticancer treatment. Overall, these findings support the idea of an integrated circulating miRNA-inflammation axis in gastrointestinal cancer patients, indicating that miRNA dysregulation may reflect additional biological aspects of the tumor–host catabolic cross-talks.

This interpretation is supported by the inflammatory profile observed in our cohort. The significant elevation of IL-6, IL-8, IL-10, and TNF-α in cancer patients compared with controls aligns with the broader tumor–host cytokine network driving CC [13].

Accordingly, in our study, the miRNA-cytokine correlations provide a biological bridge between systemic inflammation and the circulating miRNA profile, suggesting that miRNA dysregulation may be partly shaped by the inflammatory milieu that sustains cancer-associated tissue wasting.

Among the miRNAs analyzed, circulating miR-15b was remarkably higher in patients with gastrointestinal cancer compared to controls. This supports the existing evidence that miR-15b is commonly upregulated across various gastrointestinal malignancies [14]. Consistent with findings from multiple tumor types, our site-specific analysis showed increased levels in both colorectal and gastric cancer groups. However, the trend was not statistically significant in the pancreatic cancer group, likely due to its smaller sample size. Mechanistically, miR-15b has been shown to operate at the interface of inflammatory and apoptotic signaling, acting as a key mediator of inflammation-induced cancer. It exerts its effects mainly by targeting the NF-κB signaling pathway through negative regulation of NF-κB1 and one of its kinase complexes, IKK-α [15]. Because NF-κB regulates multiple components of the inflammatory response and has been shown to exert both pro- and anti-inflammatory functions [16], this dual relationship could plausibly explain the positive correlations we observed between miR-15b and both TNF-α and IL-10. Along this line, although miR-15b did not differ between cachectic and non-cachectic patients and was not selectively associated with the combined low-SM/CC phenotype, its elevation in our setting may reflect systemic inflammatory activation rather than a muscle-specific catabolic signal.

miR-21 emerged as the most robustly and consistently upregulated miRNA across all miRNAs tested, with levels significantly higher in gastrointestinal cancer patients than in controls. This result aligns with an extensive body of literature identifying miR-21 as one of the most reproducibly dysregulated miRNAs in gastrointestinal malignancies [17]. In the tumor-site analysis, miR-21 was elevated in all three subgroups compared with controls. Notably, levels were significantly higher in pancreatic than in colorectal cancer patients. Although based on a small subgroup and therefore purely exploratory, this observation raises the hypothesis of the more aggressive biological profile and greater inflammatory burden of pancreatic adenocarcinoma, consistent with studies reporting elevated circulating miR-21 as a poor prognostic factor in this tumor type [18]. This hypothesis requires confirmation in larger cohorts. When patients were stratified by cachexia status, SM mass, or their combination, miR-21 remained uniformly elevated relative to controls, with no significant differences among subgroups. This pattern contrasts with previous reports describing serum miR-21 as an independent predictor of low muscle mass in colorectal cancer [19]. This discrepancy may reflect the early-stage, treatment-naïve nature of our cohort, in which the inflammatory burden may not yet have translated into measurable structural muscle depletion. The consistent elevation across all clinical strata, together with positive correlations between miR-21 and IL-6, IL-8, and TNF-α, may suggest that its upregulation is primarily driven by the tumor-associated inflammatory milieu. This interpretation is further supported by experimental evidence indicating that miR-21 can actively modulate inflammatory signaling pathways and contribute to cytokine production, potentially amplifying the reciprocal interaction between tumor progression and systemic inflammation [20]. Therefore, the association between miR-21 and muscle depletion reported in more advanced cancer populations may become apparent only when inflammation and catabolism reach sufficient intensity to translate into overt structural muscle loss. Taken together, miR-21 may reflect a broad cancer-associated inflammatory state rather than a selective marker of cachexia or CT-defined SM depletion.

The most clinically relevant finding of our study concerns miR-29b. Our results showed significantly higher levels in patients with gastrointestinal cancer compared to controls. Elevated circulating levels have been reported in several solid tumor settings and are associated with adverse clinical outcomes [21,22], a pattern consistent with our findings. This increase was consistently observed across colorectal, gastric, and pancreatic cancer subgroups. Notably, pancreatic cancer patients showed the highest levels. This observation may reflect the greater inflammatory and catabolic burden typically associated with this tumor type. However, due to the limited subgroup size, this observation is purely exploratory and requires testing in larger cohorts. miR-29b levels were significantly higher in cachectic than in non-cachectic patients, and this elevation persisted in the combined cachexia/muscle mass stratification, with the highest levels observed in patients with concomitant low SM and cachexia. By contrast, when CT-derived SM was considered alone, miR-29b was elevated in both low- and high-SM patients compared with controls, without significant differences between SM subgroups. This pattern suggests that circulating miR-29b is more closely associated with the systemic catabolic milieu underlying CC than with reduced SM mass alone. Indeed, the additional increase observed in patients with concomitant low SM and CC supports the hypothesis that structural muscle depletion amplifies an already established cachectic process rather than representing the primary determinant of miR-29b dysregulation. This interpretation aligns with experimental evidence demonstrating that miR-29b is induced by multiple catabolic stimuli, including CC, and may function as a common downstream effector of SM atrophy rather than a simple marker of muscle loss [23]. Consistent with this, miR-29b was the only miRNA among those tested to retain an independent association with CC after adjustment for age, sex, and tumor type, supporting its potential relevance as a circulating marker of the cachectic phenotype in gastrointestinal cancer. Accordingly, the positive correlations observed between miR-29b and IL-6, IL-8, IL-10, and TNF-α may indicate that inflammatory activation contributes to miR-29b induction in our cohort. This is biologically and mechanistically plausible, as miR-29b promotes SM atrophy by directly targeting the IGF-1/PI3K pathway, thereby attenuating AKT signaling and facilitating FOXO-dependent activation of the ubiquitin-proteasome system, including atrogin-1 and MuRF1 [24]. In line with this interpretation, recent data in metastatic colorectal cancer showed that patients with cachexia and systemic inflammation displayed a distinct circulating miRNA profile, including miR-29b among the altered miRNAs, with enrichment of pathways related to immune responses, extracellular matrix organization, and proteolytic activity [25].

For miR-29a, we did not observe significant modulation across the analyzed stratifications. Unlike miR-29b, miR-29a was not associated with cachexia status, muscularity levels, or cytokine profile. This was unexpected, given that elevated circulating miR-29a has been reported in colorectal cancer patients and proposed as a tool for early detection [25,26]. Although the reasons for this discrepancy remain unclear and require further investigation, our findings indicate that miR-29a is not involved in the inflammation-associated cachectic signature identified in the present cohort. However, this negative finding is informative, as it points to a functional divergence between the two principal members of the miR-29 family within the cachectic phenotype, despite their close sequence homology and overlapping predicted targets. Similar differences between miR-29 family members have been reported in experimental models of CC, where miR-29a and miR-29b exhibited distinct patterns of regulation during SM wasting [7].

miR-133a levels were lower in cancer patients than in controls, consistent with previous evidence of reduced miR-133a expression in colorectal cancer [27] and with studies reporting miR-133a downregulation in gastric cancer, where lower expression has been associated with tumor progression and clinicopathological aggressiveness [28]. In our tumor-site analysis, miR-133a reduction was significant in the colorectal and gastric cancer subgroups, whereas the lack of significance in pancreatic cancer should be interpreted cautiously given the very small subgroup size.

Across the CC stratification, miR-133a was reduced in both cachectic and non-cachectic patients compared with controls, with no selective difference between the two cancer subgroups. However, when CT-derived body composition was considered, the reduction was confined to patients with low SM and to the combined low-SM/CC phenotype. This pattern suggests that circulating miR-133a is more closely associated with structural muscle depletion than with cachexia status alone. This interpretation is consistent with recent serum RNA sequencing data from metastatic colorectal cancer, in which miR-133a was the only miRNA significantly downregulated in cachectic versus non-cachectic patients [25]. The preferential association of miR-133a with CT-defined muscle depletion suggests that this myomiR primarily reflects alterations in SM integrity rather than the systemic manifestations of CC. This interpretation is supported by miR-133a’s biological role as a muscle-enriched myomiR involved in myoblast proliferation, differentiation, and maintenance of skeletal muscle homeostasis [8]. Among all the myomiRs analyzed, miR-133a showed the strongest inverse correlations with IL-6, IL-8, and IL-10. Although these associations do not demonstrate causality, they support the hypothesis that progressive muscle deterioration and systemic inflammation evolve in parallel during cancer progression. In this context, the decline in circulating miR-133a may not simply reflect reduced muscle mass but rather the loss of metabolically active and regenerative SM within an increasingly inflammatory environment. This interpretation is consistent with experimental evidence showing that inflammatory cytokines impair myogenic differentiation and regenerative capacity, ultimately promoting muscle remodeling and wasting [29,30].

miR-206 was significantly reduced in patients with gastrointestinal cancer compared with controls, consistent with previous studies reporting decreased miR-206 expression in gastrointestinal malignancies [31,32]. When tumor subgroups were analyzed separately, miR-206 remained significantly reduced in gastric and pancreatic cancer, whereas no significant reduction was observed in colorectal cancer. Given the limited number of gastric and pancreatic cancer patients, these findings should be interpreted as exploratory. Across the CC stratification, miR-206 was significantly reduced only in cachectic patients compared with controls, whereas no significant differences were observed between non-cachectic patients and controls or between cachectic and non-cachectic patients. CT-derived muscularity alone did not discriminate circulating miR-206 levels. When CC and body composition were analyzed together, the reduction was again confined to the low-muscularity/CC phenotype. Mechanistically, miR-206 is an SM-specific myomiR that regulates key processes of muscle differentiation and homeostasis [33] and has been reported to decrease in muscle wasting, including sarcopenia and other muscle-wasting disorders [34,35]. However, despite this canonical muscle-restricted role, our data show that circulating miR-206 tracked more closely with the presence of CC than with CT-derived SM loss, pointing to a predominant influence of the systemic catabolic state underlying CC over muscle quantity *per se*. Consistent with the possibility that miR-206 expression might not simply reflect a passive readout of muscle quantity, its weak inverse correlations with IL-6 and IL-10, together with the lack of association with IL-8 and TNF-α, may indicate that its regulation is linked to specific inflammatory pathways involved in cachexia. Indeed, both IL-6 and IL-10 converge on STAT3-dependent signaling, a pathway critically involved in cancer-associated muscle catabolism [36,37], whereas IL-8 and TNF-α predominantly activate distinct inflammatory cascades, such as NF-κB-related signaling [38,39]. Although the underlying mechanisms remain to be established, this signaling framework may partly explain the selective inverse association of circulating miR-206 with IL-6 and IL-10 observed in our cohort. Supporting this interpretation, a bidirectional relationship between miR-206 and IL-6/STAT3 has been described in other tumor contexts, in which IL-6 suppresses miR-206 maturation and miR-206, in turn, inhibits IL-6/STAT3 signaling [40]. Although this mechanism has not been demonstrated in CC, it provides biological plausibility for the hypothesis that persistent activation of cachexia-associated inflammatory signaling may contribute to the reduction in circulating miR-206 observed in our cohort.

Levels of miR-486 were significantly lower in patients with gastrointestinal cancer than in controls, consistent with previous reports of reduced miR-486 expression in gastrointestinal malignancies [41,42]. When tumor subgroups were analyzed separately, this reduction was confirmed in colorectal and pancreatic cancers, whereas no significant difference was observed in gastric cancer. Although limited by the small sample size, these exploratory findings may suggest that miR-486 regulation differs across gastrointestinal tumor types, in line with previous evidence indicating that miR-486 displays tissue- and context-dependent regulation across different pathological conditions [43]. Surprisingly, circulating miR-486 did not discriminate patients by CC, CT-derived muscularity, or their combined phenotype. These results partially differ from previous studies reporting that circulating miR-486 is positively associated with the SM index and is significantly reduced in sarcopenic individuals [44], as well as from preclinical evidence showing reduced miR-486 expression in the SM of cancer-bearing cachectic mice [12]. This apparent discrepancy may indicate that the generalized reduction in circulating miR-486 across all patient subgroups may reflect an early cancer-associated alteration that precedes the onset of CC or measurable SM depletion. Mechanistically, miR-486 supports SM homeostasis by targeting PTEN and FOXO1-related signaling, thereby favoring PI3K/AKT activity and limiting atrophy-related transcriptional programs [45]. Therefore, although circulating miR-486 is biologically linked to anabolic signaling, its systemic reduction in our cohort may primarily reflect the cancer-related dysregulation of this pathway rather than the severity of SM wasting. The weak inverse correlation with IL-8 may further support this interpretation, suggesting that inflammatory stress may contribute to miR-486 downregulation without being sufficient to determine the cachectic or low-muscularity phenotype. Although speculative, these findings may explain why circulating miR-486 was consistently reduced across the entire gastrointestinal cancer cohort, yet showed limited ability to discriminate body composition phenotypes, suggesting that tissue-specific alterations associated with SM wasting may not be fully captured by circulating miR-486 in treatment-naïve gastrointestinal cancer patients. Therefore, larger multicenter longitudinal studies integrating tissue analyses and functional experimental models are warranted to validate these findings, clarify the biological origin and mechanistic role of the identified miRNAs.

Several limitations of the present study should be acknowledged. First, the single-center, observational, cross-sectional design and the lack of longitudinal follow-up preclude causal inference and do not allow assessment of whether circulating miRNA alterations precede or follow the onset of CC and body composition changes. Second, miRNAs were measured exclusively in plasma without paired SM biopsies, preventing identification of their tissue origin and direct comparison of circulating and tissue expression. Moreover, the absence of functional validation precludes conclusions regarding the mechanistic involvement of the identified miRNAs in SM wasting. Third, the relatively small sample size, particularly for the gastric and pancreatic cancer subgroups, limits statistical power and requires cautious interpretation of the exploratory subgroup analyses. Moreover, the predominance of colorectal cancer patients (87% of the cohort) limits the generalizability of our findings to other gastrointestinal malignancies, which were only minimally represented in this study. Furthermore, CT imaging was unavailable in the control group, precluding direct comparison of body composition between patients and control subjects. Additionally, given the exploratory design of the study, no adjustment for multiple comparisons was performed. Therefore, the findings should be interpreted as hypothesis-generating and require confirmation in independent cohorts. In addition, weight loss assessment was based on self-reported body weight measurements, which may be subject to recall bias. Finally, the control group was significantly younger than the gastrointestinal cancer cohort, and although age was included as a covariate in the multivariable analysis of CC, the comparisons between patients and controls were not age-adjusted. Therefore, a residual effect of age on circulating miRNA levels cannot be completely excluded. Future multicenter longitudinal studies integrating paired tissue analyses and mechanistic experimental studies are under way to validate these findings and further clarify the biological origin and functional role of circulating miRNAs in CC.

## 5. Conclusions

Circulating miRNAs showed distinct associations with systemic inflammation, CC, and SM status in treatment-naïve patients with gastrointestinal cancer. Among the analyzed miRNAs, miR-29b was more closely associated with the cachectic phenotype, whereas miR-133a appeared preferentially associated with SM depletion. The selective association of miR-29b with CC, together with its correlations with inflammatory cytokines and its further increase in patients with concomitant low SM mass and CC, may suggest that this miRNA reflects an early circulating signal of inflammation-associated catabolic reprogramming rather than reduced SM mass alone. These findings support the hypothesis that circulating miRNA alterations reflect distinct components of the host response to cancer and warrant further investigation in larger prospective studies.

## Figures and Tables

**Table 1:** Clinical and demographic characteristics of the study population.

	Controls *n* = 15	Gastrointestinal Cancer Patients *n* = 54	*p*-Value
**Female, n (%)**	7 (47)	21 (39)	
**Age (years), median (IQR)**	54.0 (51.0; 66.0)	73.0 (64.0; 78.0)	*p = 0.003*
**BMI (kg/m^2^), mean ± SD**	23.0 ± 3.2	25.0 ± 4.3	*p = 0.473*
**Weight loss in previous 6 months, n (%)**	/	24 (44)	
**SMA (cm^2^), mean ± SD**	/	113.7 ± 40.7	
**SMI (cm^2^/m^2^), mean ± SD:**			
**Male**	/	50.98 ± 8.1	
**Female**	/	39.44 ± 6.6	
**Blood parameters:**			
**C-reactive protein, mg/dL**	1.1 (0.2; 1.6)	3.1 (0.6; 8.6)	*p = 0.151*
**Haemoglobin, g/dL**	11.2 ± 1.2	12.3 ± 2.02	*p = 0.826*
**Albumin, g/dL**	3.1 ± 0.6	3.4 ± 0.6	*p = 0.313*
**Creatinine, mg/dL**	1.1 (0.7; 1.1)	0.9 (0.7; 1.3)	*p = 0.494*
**Main comorbidities/habits:**			
**Diabetes mellitus, n (%)**	2 (13%)	10 (19)	
**Dyslipidemia, n (%)**	1 (7%)	12 (22)	
**Smoking, n (%)**	3 (20%)	8 (15)	
**Tumor site:**			
**Colon, n (%)**		47 (87%)	
**Stomach, n (%)**		4 (7%)	
**Pancreas, n (%)**		3 (6%)	
**Stage of the disease, n (%)**			
**I–II**		33 (61%)	
**III–IV**		21 (39%)	

Abbreviations: BMI, body mass index; CRP, C-reactive protein; SMA, skeletal muscle area; SMI, skeletal muscle index; IQR, interquartile range; SD, standard deviation.

**Table 2:** Multivariable logistic regression model assessing the association of circulating miR-29b with cancer cachexia.

Variable	B	*p*-Value	OR	95% CI
Age	0.038	0.216	1.039	0.978–1.103
Gender	0.021	0.974	1.021	0.293–3.560
Tumor type, non-CRC vs. CRC	−1.656	0.225	0.191	0.013–2.774
miR-29b	2.391	0.007	10.926	1.933–61.756

Abbreviations: CI, confidence interval; CRC, colorectal cancer; OR, odds ratio.

## Data Availability

The original contributions presented in this study are included in the article/Supplementary Materials. Further inquiries can be directed to the corresponding author.

## Supplementary Materials

The following supporting information can be downloaded at https://www.mdpi.com/article/10.3390/cancers18213509/s1: Figure S1: Circulating miRNA profile according to tumor type in patients with gastrointestinal cancer and controls; Table S1: Spearman’s correlation analysis between circulating miRNAs and cytokines in gastrointestinal cancer patients and controls.

## Abbreviations

BMI	Body Mass Index
CC	Cancer Cachexia
CT	Computed Tomography
HMM	High Muscle Mass
IL-	Interleukin-
L3	Third Lumbar Vertebra
LMM	Low Muscle Mass
miRNA/miR	microRNA
SM	Skeletal Muscle
SMA	Skeletal Muscle Area
SMI	Skeletal Muscle Index
TNF-α	Tumor Necrosis Factor-alpha
