# Supplementary material for: Circulating microRNAs Capture Inflammatory and Muscle Wasting Phenotypes in Patients with Gastrointestinal Cancer
Source: Cancers (Basel). 2026 Oct 31;18(21):3509. doi: 10.3390/cancers18213509 (PMC13762101; doi:10.3390/cancers18213509)
Supplement: Supplementary file 1 [file cancers-18-03509-s001.zip › cancers-4617817-Supplementary Figures miR TM.pdf]

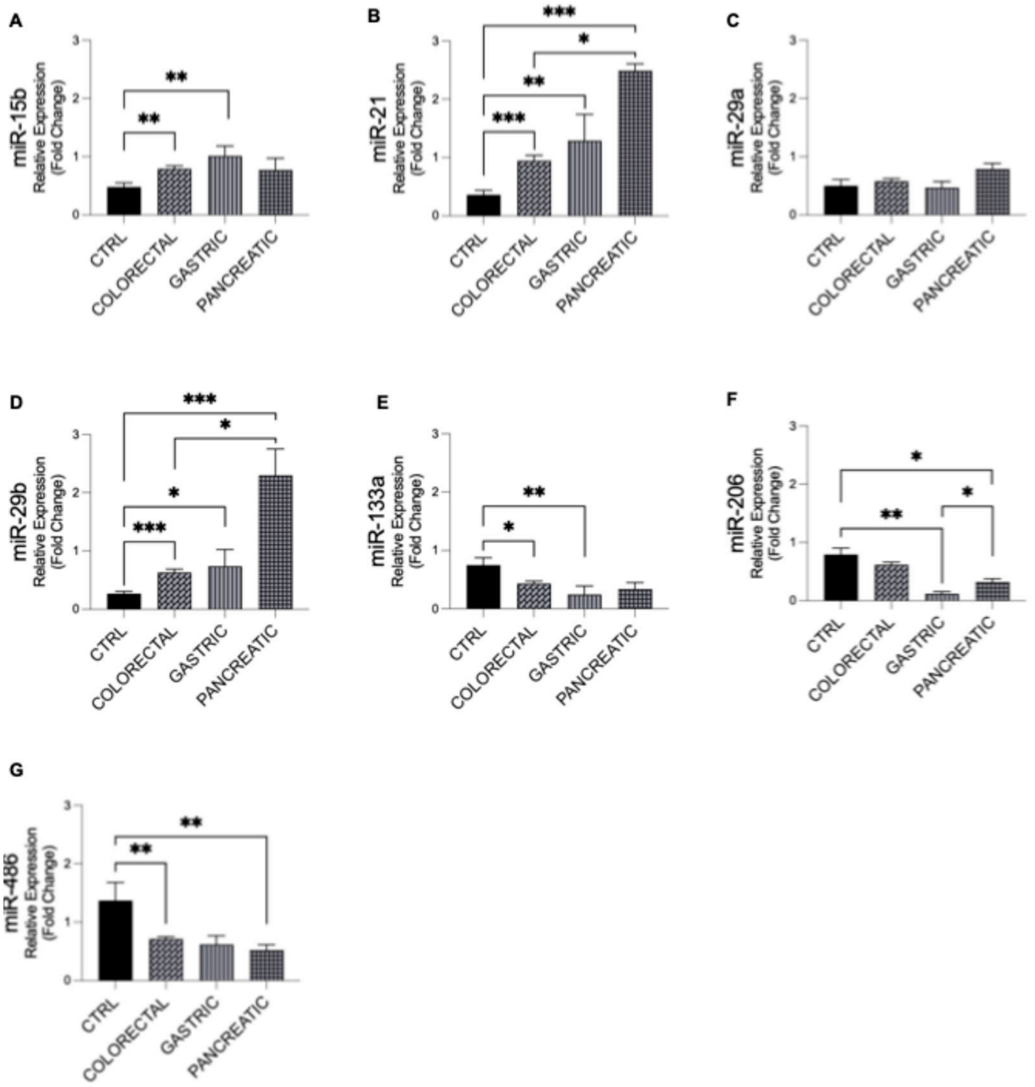

Supplementary Figure S1. Circulating miRNA profile according to tumor type in patients with gastrointestinal cancer and controls. Circulating miRNA levels were analyzed in patients with colorectal cancer (n = 47), gastric cancer (n = 4), pancreatic cancer (n = 3), and controls (n = 15). (**A**) With respect to controls, miR-15b levels were significantly increased in colorectal and gastric cancer patients (p=0.006 and p = 0.009, respectively). (**B**) miR-21 levels were significantly increased in colorectal (p < 0.001), gastric (p = 0.010), and pancreatic (p < 0.001) cancer patients compared with controls, with a significant modulation between colorectal and pancreatic cancer patients (p = 0.022). (**D**) miR-29b levels were significantly increased in colorectal (p < 0.001), gastric (p = 0.015), and pancreatic (p < 0.001) cancer patients vs controls, with a significant modulation between colorectal and pancreatic cancer patients (p = 0.017). (**E**,**G**) miR-133a levels were significantly reduced in colorectal (p = 0.017) and in gastric (p = 0.007) cancer patients compared with controls, while miR-486 levels were significantly lower in colorectal (p = 0.002) and in pancreatic (p = 0.009) cancer patients vs the control group. (**F**) miR-206 levels were significantly decreased in gastric (p = 0.001) and pancreatic (p = 0.037) cancer patients vs controls, with a significant modulation between gastric and pancreatic cancer patients (p = 0.014). (**C**) No significant differences were observed for miR-29a.

Data are expressed as fold change and are presented as mean ± SEM. One-way ANOVA or Kruskal-Wallis test was used for normally distributed or non-normally distributed data, respectively. * p ≤ 0.05, ** p ≤ 0.01, *** p ≤ 0.001.
