# Supplementary material for: Circulating microRNAs Capture Inflammatory and Muscle Wasting Phenotypes in Patients with Gastrointestinal Cancer
Source: Cancers (Basel). 2026 Oct 31;18(21):3509. doi: 10.3390/cancers18213509 (PMC13762101; doi:10.3390/cancers18213509)
Supplement: Supplementary file 1 [file cancers-18-03509-s001.zip › cancers-4617817-Supplementary Table miR TM.pdf]

Table S1. Supplementary. Spearman's correlation analysis between circulating miRNAs and cytokines in gastrointestinal cancer patients and control group.

	IL-6 (pg/mL)	IL-8 (pg/mL)	IL-10 (pg/mL)	TNFα (pg/mL)
**miR-15b**	*r* = 0.182<br>*p* = 0.134	*r* = 0.155<br>*p* = 0.202	*r* = 0.383<br>*p* = 0.001 *	*r* = 0.347<br>*p* = 0.003 *
**miR-21**	*r* = 0.330<br>*p* = 0.006 *	*r* = 0.226<br>*p* = 0.017 *	*r* = 0.212<br>*p* = 0.081	*r* = 0.298<br>*p* = 0.013 *
**miR-29a**	*r* = 0.129<br>*p* = 0.289	*r* = 0.086<br>*p* = 0.480	*r* = 0.219<br>*p* = 0.071	*r* = 0.070<br>*p* = 0.558
**miR-29b**	*r* = 0.413<br>*p* <0.001 *	*r* = 0.403<br>*p* < 0.001 *	*r* = 0.277<br>*p* = 0.021 *	*r* = 0.259<br>*p* = 0.032 *
**miR-133a**	*r* = −0.301<br>*p* = 0.012 *	*r* = −0.276<br>*p* = 0.022 *	*r* = −0.383<br>*p* = 0.001 *	*r* = −0.139<br>*p* = 0.256
**miR-206**	*r* = −0.334<br>*p* = 0.05 *	*r* = −0.037<br>*p* = 0.763	*r* = −0.288<br>*p* = 0.016 *	*r* = −0.146<br>*p* = 0.232
**miR-486**	*r* = −0.209<br>*p* = 0.085	*r* = −0.266<br>*p* = 0.027 *	*r* = −0.195<br>*p* = 0.108	*r* = −0.229<br>*p* = 0.058

Abbreviations: IL, interleukin; TNF-α, tumor necrosis factor-alpha.
